# MTGEA: A Multimodal Two-Stream GNN Framework for Efficient Point Cloud and Skeleton Data Alignment

**DOI:** 10.3390/s23052787

**Published:** 2023-03-03

**Authors:** Gawon Lee, Jihie Kim

**Affiliations:** Department of Artificial Intelligence, Dongguk University, 30 Pildong-ro 1 Gil, Seoul 04620, Republic of Korea

**Keywords:** human activity recognition, mmWave radar, Kinect V4 sensor, point clouds, skeleton data, multimodal, two stream, attention mechanism

## Abstract

Because of societal changes, human activity recognition, part of home care systems, has become increasingly important. Camera-based recognition is mainstream but has privacy concerns and is less accurate under dim lighting. In contrast, radar sensors do not record sensitive information, avoid the invasion of privacy, and work in poor lighting. However, the collected data are often sparse. To address this issue, we propose a novel Multimodal Two-stream GNN Framework for Efficient Point Cloud and Skeleton Data Alignment (MTGEA), which improves recognition accuracy through accurate skeletal features from Kinect models. We first collected two datasets using the mmWave radar and Kinect v4 sensors. Then, we used zero-padding, Gaussian Noise (GN), and Agglomerative Hierarchical Clustering (AHC) to increase the number of collected point clouds to 25 per frame to match the skeleton data. Second, we used Spatial Temporal Graph Convolutional Network (ST-GCN) architecture to acquire multimodal representations in the spatio-temporal domain focusing on skeletal features. Finally, we implemented an attention mechanism aligning the two multimodal features to capture the correlation between point clouds and skeleton data. The resulting model was evaluated empirically on human activity data and shown to improve human activity recognition with radar data only. All datasets and codes are available in our GitHub.

## 1. Introduction

As the world population ages, older persons are a growing group in society. According to World Population Prospects 2019 (United Nations, 2019), by 2050, the number of persons aged 65 years or over globally will surpass those aged 15–24. In addition to this, single-person households have increased tremendously in the last few years due to societal changes. With these population changes, home care systems have emerged as a promising venue of intelligent technologies for senior and single-person households. In addition, the recent COVID-19 pandemic has further increased the importance of developing home care systems. The current mainstream home care systems are based on cameras [1]; however, people can feel uncomfortable being recorded by cameras and hence might refuse to be monitored by camera-based techniques. The biggest problem is the invasion of privacy. If the personal data recorded by the camera is leaked, it may have devastating consequences. There is also a problem with the accuracy of the camera being affected by the lighting and its placement. Consequently, alternative approaches to home care are needed.

With the advances in Frequency-Modulated Continuous Wave (FMCW) mmWave technology, human activity recognition by mmWave radar sensors has recently attracted significant attention. A radar sensor can collect 3D coordinates called point clouds while emitting and absorbing radio waves to and from objects. Moreover, depending on the hardware or data collection tool type, other data (e.g., range and velocity) can be captured simultaneously. A radar sensor also does not require a strict environment setting. In other words, it works correctly even in poor lighting and with poor camera placement. Because a radar sensor does not record personal information as an image or video, the issue of invasion of privacy is significantly reduced. However, radar produces sparse point clouds due to the radar sensor’s radio wavelength and inherent noise. Many researchers have devoted effort to processing sparse radar data [2,3,4,5] and have thus devised voxelization. Voxelization is a method that converts point clouds into voxels with constant dimensions, which researchers decide empirically. Singh et al. [6] voxelized point clouds with dimensions 60 × 10 × 32 × 32 (depth = 10) and then fed them into a set of classifiers. Although voxelization is a well-known pre-processing method, it is inefficient, as researchers must decide the dimensions empirically. Using upsampling techniques to deal with the sparsity of the point clouds is another popular method. Palipana et al. [7] resampled the number of points to achieve a fixed number. They used Agglomerative Hierarchical Clustering (AHC) for upsampling. The AHC algorithm adds a cluster’s centroid as a new point after clustering the point clouds.

Another popular sensor is the Microsoft Kinect [8,9], which provides various data such as RGB videos, depth sequences, and skeleton information. In recent years, many studies have taken advantage of skeleton data because of their robustness to human appearance change as well as illumination. Hence, plenty of related skeleton data (e.g., NTURGB+D [10] and NTU-RGB+D 120 [11]) has been collected and used. Rao et al. [12] proposed learning the pattern invariance of actions using a momentum Long Short-Term Memory (LSTM) after seven augmentation strategies to boost action recognition accuracy via 3D skeleton data. To overcome the sparsity of point clouds, we propose exploiting this skeleton data in radar-based recognition, and we designed a multimodal framework that can effectively combine point clouds with useful skeleton information.

Depth video recordings gathered using Kinect were also utilized for human activity recognition. In the [13], the authors pre-processed the dataset recorded by depth cameras. To avoid misleading context, separating poses and removing context were needed. However, the opportunities for learning more from the background rather than a real person’s data remain, and recorded videos have privacy issues.

In the case of wearable sensors, Wozniak et al. [14] identify the user’s body position using wearable sensor data from various body parts, such as the ankle, wrist, waist, and chest. They have decided only two sensors are enough to obtain up to 100% accuracy in a thorough examination. Although proposed models in [14] achieved 99.89% accuracy rates, wearable devices which touch body parts, such as the chest, during data collection, can be quite cumbersome in actual use, especially for children or elderly people.

Various multimodal frameworks that take advantage of data from multiple sources have already been studied. As such, fusion strategies for combining multimodal features have been devised. These include concatenation [15], attention mechanisms [16], and a simple weight-sum manner [17].

Based on these results, this paper proposes a novel Multimodal Two-stream GNN Framework for Efficient Point Cloud and Skeleton Data Alignment (MTGEA) to improve human activity recognition with radar data. The proposed framework utilizes spatial temporal graph convolutional networks (ST-GCNs) as graph neural networks (GNNs), which can effectively capture both temporal and spatial features. Three upsampling techniques were used to address the sparsity of point clouds. In addition, unlike previous work, which uses the single-modal framework, we constructed a multimodal framework with skeletal data so that reliable features could be obtained. While strict one-to-one mapping is difficult due to the different types of environmental settings, in the proposed model, the point clouds and skeleton data can be used together as 3D coordinates. Based on the embedded representations generated from applying ST-GCN to both data, we incorporated an attention mechanism in aligning the point clouds and skeleton data and attained structural similarity and accurate key features from the two datasets. Then, the aligned features and embedded features of point clouds were concatenated to form the final classification decision. For the reasoning of human activity recognition, we used the radar data only, with the Kinect part frozen. We evaluated MTGEA empirically with seven human activity data, including falling. All data were collected by mmWave radar and Kinect v4 sensors simultaneously. In summary, our main contributions are as follows:

- We propose a novel MTGEA. Our major contribution is presenting a new approach for incorporating accurate Kinect skeletal features into the radar recognition model, enabling human activity recognition using sparse point clouds alone without having to use the Kinect stream during reasoning;
- We propose skeleton data with an attention mechanism as a tool for generating reliable features for the multimodal alignment of point clouds. We also utilize three upsampling techniques to address the sparsity of radar point clouds;
- We provide a new point cloud and skeleton dataset for human activity recognition. All data simultaneously collected by mmWave radar and Kinect v4 sensors are open source, along with the entire code and pre-trained classifiers.

## 2. Related Works

Early research on detecting human actions usually used images. Ogundokun et al. [18] proposed a deep convolutional neural network (DCNN) framework for human posture classification. They chose DCNN for deriving abstract feature maps from input data. However, the pixels of images and image sequences have various backgrounds, so features should be carefully extracted due to the risk of privacy invasion.

So, in the case of radar sensors, most researchers focused on pre-processing sparse point clouds. One of the popular methods was voxelization. Sengupta et al. [19] presented mmPose-NLP, an mmWave radar-based skeletal keypoint inspired by natural language processing (NLP). In their study, point clouds were first pre-processed through voxelization. Authors regarded this method as a process similar to the tokenization of NLP. The mmPose-NLP architecture was applied to predict the voxel indexes, corresponding to 25 skeleton key points. To measure the accuracy of the proposed system, the authors used the Mean Absolute Error (MAE) metric. However, voxelization pre-processing methods, which usually require a fixed shape, are augmented sequences. In the case of point clouds, Palipana et al. [7] proposed an upsampling method to expand sparse point clouds. They used AHC for upsampling until they achieved a fixed number of point clouds. In the AHC algorithm, all point clouds formed clusters first, and each cluster’s centroid was added to the point clouds as a new point. We provide more detailed information regarding the AHC algorithm in Section 3.2.

In [20], a pre-trained model based on two consecutive convolution neural networks (CNNs) was used to extract reliable features in skeleton form from sparse radar data. Then, the GNN-based model was applied for classification. It achieved above 90% accuracy on the MMActivity dataset [6]. However, two-phase flow models such as this can be inefficient.

In this paper, we utilized the two-stream multimodal framework and alignment method to exploit an accurate skeleton dataset from Kinect. Many previous researchers have devised various alignment methods for proper feature fusion. Yang et al. [17] built a shallow graph convolutional network with a two-stream structure for bone and joint skeleton data and proposed a weight-sum manner to obtain the final prediction. This method requires a lower computational cost and is relatively simple. Concatenation is one of the popular methods for feature fusion. Pan et al. [21] proposed a Variational Relational Point Completion Network (VRCNet) to construct complete shapes for partial point clouds. VRCNet had two consecutive encoder–decoder sub-networks named probabilistic modeling (PMNet) and relational enhancement (RENet). In the PMNet, the concatenation of coarse complete point clouds and incomplete point clouds occurred, which led to the generation of the overall skeletons. Weiyao et al. [15] proposed a multimodal action recognition model based on RGB-D and adopted skeleton data as the multimodal data. The proposed network consisted of GCN and CNN. The GCN network took the skeletal sequence, and R (2+1)D based on the CNN network architecture took the RGB video. Then, the outer product of two compressed features was obtained to make the final classification decision. Zheng et al. [16] designed a Multimodal Relation Extract Neural Network with Efficient Graph Alignment (MEGA). To identify textual relations using visual clues, MEGA utilized visual objects in an image and textual entities in a sentence as multimodal data. The authors conducted experiments using the MNRE dataset, demonstrating that the alignment of visual and textual relations by attention could improve the relation extraction performance. In this paper, we created a skeleton and point cloud dataset and used these sensor data as multimodal data. Then, we utilized an attention mechanism to integrate these two features to assist in generating more reliable features.

## 3. Methodology

### 3.1. Subsection Experimental Environments and Dataset

**Figure 1:**
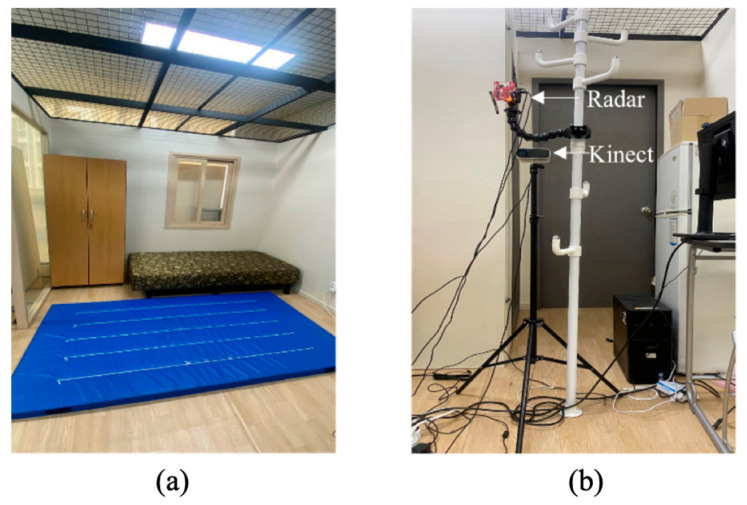
Experimental environments for the DGUHA dataset. (**a**) Data collection environments, and (**b**) Data collection setup.

**Figure 2:**
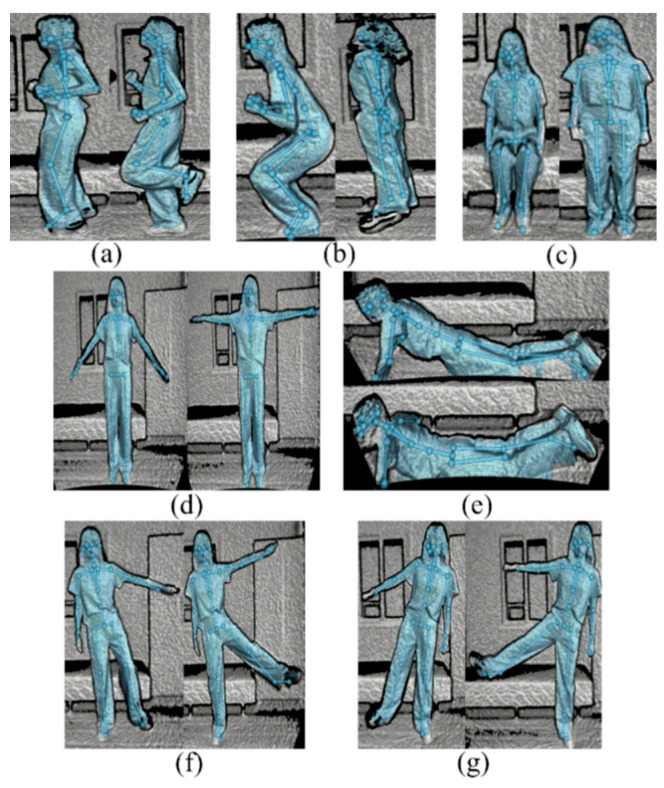
The DGUHA dataset collected in our experiments. (**a**) Running, (**b**) Jumping, (**c**) Sitting down and standing up, (**d**) Both upper limb extension, (**e**) Falling forward, (**f**) Right limb extension, and (**g**) Left limb extension.

Training and test data were collected following a study protocol approved by the Institutional Review Board of Dongguk University (Approval number: DUIRB-202104-04). We recruited 19 subjects to collect the new dataset, the DGUHA (Dongguk University Human Activity) dataset, which includes both point cloud and skeleton data. All subjects were in their twenties (the average age was 23 years). In the environment shown in Figure 1a, each subject performed seven movements: running, jumping, sitting down and standing up, both upper limb extension, falling forward, right limb extension, and left limb extension, as illustrated in Figure 2 (This figure was captured from the authors and thus did not require approval from IRB). All of the subjects performed each activity for about 20 s. Including break time, data collection was performed for 1 h, and all activities were repeated approximately 5–6 times during this time. We utilized an mmWave radar sensor and Microsoft Kinect v4 sensor to collect the data.

In the case of the mmWave radar sensor, TI’s IWR1443BOOST radar (Texas Instruments, city and country: Dallas, TX, USA), which includes four receivers and three transmitters, was used. It is based on FMCW, of which a chirp signal is a fundamental component. After transmitters emit an FMCW signal, receivers detect objects in a 3D plane by measuring the delay time according to the distance to the target as a frequency difference. The sensor was mounted parallel to the ground at a height of 1.2 m, as shown in Figure 1b. The sampling rate of the radar was 20 fps, and we collected the data using a robot operating system [22]. We stored five primary data modalities: 3D coordinates (x, y, and z in m), range, velocity, bearing angle (degrees), and intensity. The 3D coordinates are usually called point clouds.

**Figure 3:**
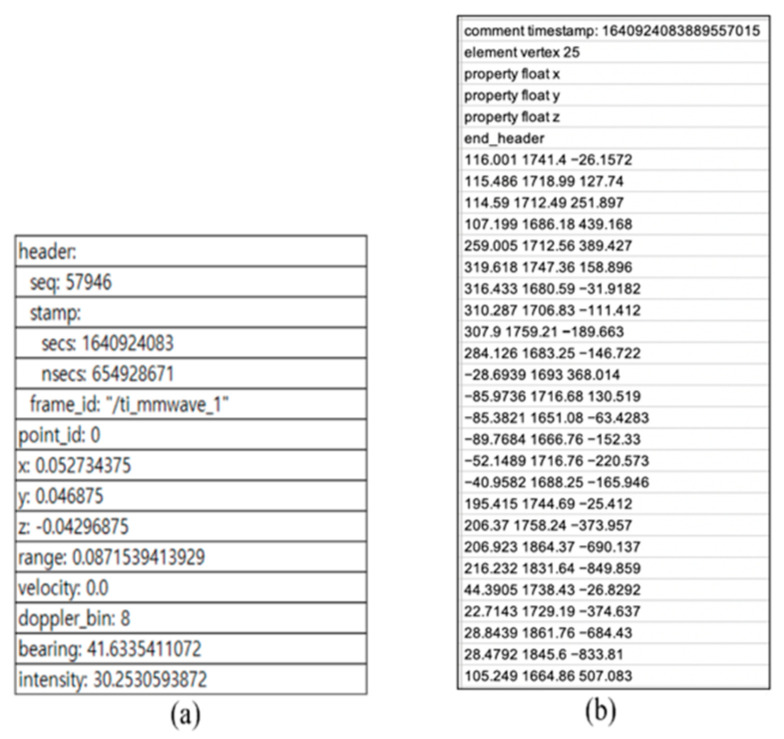
The DGUHA dataset format. (**a**) mmWave radar data format in DGUHA, and (**b**) Kinect data format in DGUHA.

The Microsoft Kinect v4 sensor was also mounted parallel to the ground at a height of 1 m, as shown in Figure 1b. A total of 25 skeleton data represented the 3D locations of 25 major body parts: spine, chest, neck, left shoulder, left elbow, left wrist, left hand, left hand tip, left thumb, right shoulder, right elbow, right wrist, right hand, right hand tip, right thumb, left hip, left knee, left ankle, left foot, right hip, right knee, right ankle, right foot, and head. It captured skeleton data at a sampling rate of 20 fps. We collected the two datasets on Ubuntu 18.04 system simultaneously, and they were saved as a text file, as illustrated in Figure 3.

### 3.2. Data Augmentation

The sampling rates of both sensors were the same, and each activity was performed for 20 s, as mentioned in Section 3.1. Although exact one-to-one mapping was difficult due to the different types of hardware and data collection tools, the two datasets were stored at 400 frames per activity. If there were fewer than 400 frames, we replaced missing frames with the last ones. In contrast, extra frames were removed to maintain 400 frames. We randomly picked data files from each activity to check the average, median, and mode of the number of point clouds. As shown in Table 1, the point clouds were sparse. This sparsity is because of the radar sensor’s radio wavelength and inherent noise. To address the above challenge, we applied three upsampling techniques introduced in [7,12] to the point clouds.

To use the skeleton data collected from Kinect simultaneously with those from the radar sensor as multimodal data, our upsampling techniques aimed to augment the number of point clouds to 25 per frame to match the number of joints in the collected skeleton data. To augment the number of point clouds, we used the following techniques for upsampling:

(1) Zero-Padding (ZP): ZP is the simplest and most efficient of the many data augmentation methods. We padded the remaining points with zeros to obtain 25-point clouds;

(2) Gaussian Noise (GN): The GNs were generated based on the standard derivations (SDs) of the original datasets. After ZP, we added Gaussian noise *N* (0, 0.05) over point clouds according to the following formula:

(1) $$N\left(x|\mu ,\sigma^{2}\right)=\frac{1}{{\left(2\pi \sigma^{2}\right)}^{\frac{1}{2}}}\exp\left\{-\frac{1}{2\sigma^{2}}{\left(x-\mu \right)}^{2}\right\}$$

(3) Agglomerative Hierarchical Clustering (AHC): This algorithm is a bottom-up and iterative clustering approach. It consists of three steps. First, the dissimilarity between all data is calculated. Generally, Euclidean distance or Manhattan distance can be calculated. Second, the two closest data are clustered to create a class. Finally, the dissimilarity between the cluster and other data or between clusters is calculated. These three steps are repeated until all data become one cluster. Maximum, minimum, and mean can be calculated to measure the dissimilarity of the two clusters.

### 3.3. Feature Extraction Using ST-GCNs

**Figure 4:**
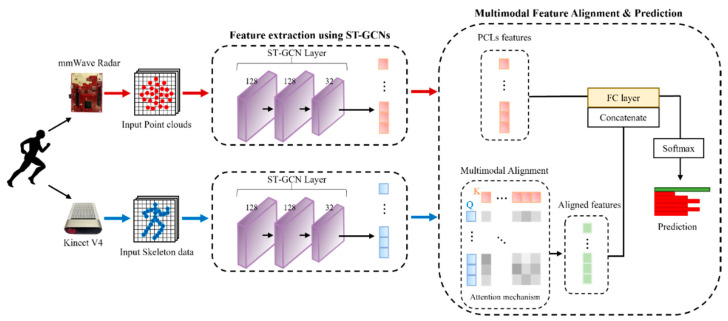
Illustration of the MTGEA. Three ST-GCN layers with the same channels were used to extract features in both point cloud and skeleton data. After passing the ST-GCN layers, features were extracted in the spatio-temporal domain from 3D coordinate data. Their features were then transformed into the matrixes Q, K, and V by three learnable matrixes, and the attention function was calculated, after which an aligned feature was obtained. The aligned and point cloud features were then concatenated and sent to the fully connected layer to form a final classification decision.

We obtained 25 point clouds through upsampling to match the skeleton data. We then used the ST-GCN architecture to acquire multimodal representation, as illustrated in Figure 4. The GNN used in the proposed MTGEA is the ST-GCN. ST-GCN achieved promising performance by utilizing a graph representation of the skeleton data [23]. In the skeleton structure, human joints can be considered a vertex or node of a graph, and connections between them can be regarded as an edge or relation of the graph. In addition to a spatial graph based on human joints, there are temporal edges connecting joints between the previous and next steps within a movement. If a spatio-temporal graph for a movement is denoted as G = (*V*, *E*), *V* denotes the set of the joints, and *E* denotes both spatial and temporal edges. The authors [23] adopted a propagation rule similar to that of GCNs [24], which is defined as follows:

(2) $$f_{out}={\hat{A}}^{-\frac{1}{2}}\left(A+I\right){\hat{A}}^{-\frac{1}{2}}f_{in}W,\;$$

where ${\hat{A}}^{ii}=\sum_{j}(A^{ij}+I^{ij})$ and W is the weight matrix. The authors also used partitioning strategies such as distance partitioning, spatial configuration partitioning, and dismantled adjacency matrix into multiple matrixes $A_{j}$, where $A+I=\sum_{j}A_{j}$. Therefore, Equation (2) is transformed into:

(3) $$f_{out}=\sum_{j}{\hat{A}}_{j}^{-\frac{1}{2}}A_{j}{\hat{A}}_{j}^{-\frac{1}{2}}f_{in}W_{j},\;$$

where ${\hat{A}}_{j}^{ii}=\sum_{k}(A_{j}^{ik})+\varepsilon$ and ε=0.001 is used to avoid empty rows in $A_{j}$. Then, the element-wise product is conducted between $A_{j}$ and M to implement the learnable edge importance weighting. M is a learnable weight matrix and is initialized as an all-one matrix. Consequently, Equation (3) is substituted with:

(4) $$f_{out}=\sum_{j}{\hat{A}}_{j}^{-\frac{1}{2}}(A_{j}⨂M){\hat{A}}_{j}^{-\frac{1}{2}}f_{in}W_{j},\;$$

where ⨂ denotes the element-wise product. In our model, the three channels, which made up the 3D coordinates, were the input. As illustrated in Figure 4, two consecutive ST-GCN layers had the same 128 channels, and the final output of the ST-GCN contained 32 channels.

### 3.4. Multimodal Feature Alignment by Attention

In the field of NLP, an attention mechanism was first introduced in [25]. This mechanism allows a decoder to find parts to pay attention to from the source sentence. We implemented an attention mechanism to align point clouds and skeleton data. Unlike previous feature fusion methods [26,27,28,29], which operate by concatenating the features or simply calculating a weight-sum, an attention mechanism can find the structural similarity and accurate key features between two features, resulting in the generation of reliable features. These reliable features can help our model address sparse point clouds and recognize human activities more accurately. The input of the attention function, (scaled dot-product attention) [30], consists of a query, a key of the dimension $d_{k}$ and values of the dimension $d_{v}.$ We set $d_{k}$ and $d_{v}$ to the same number $d_{t},$ as proposed in [16], for simplicity. Queries, keys, and values were packed into matrixes *Q*, *K*, and *V*, respectively, and the matrix of outputs was calculated as:

(5) $$Attention\left(Q,K,V\right)=softmax\left(\frac{QK^{T}}{\sqrt{d_{t}}}\right)V,\;$$

where the dot products of the query with all keys are scaled down by $d_{t}$. In practice, we projected each point cloud and skeleton data into a common t-dimensional space using an ST-GCN, achieving point cloud representation $X\in {\mathbb{R}}^{N\times d_{t}}$ and skeleton representation $Y\in {\mathbb{R}}^{N\times d_{t}}$. Then, we used three learnable matrixes $W_{q}\in {\mathbb{R}}^{d_{t}\times d_{t}}$, $W_{k}\in {\mathbb{R}}^{d_{t}\times d_{t}}$ and $W_{v}\in {\mathbb{R}}^{d_{t}\times d_{t}}$ empirically to generate the matrixes *Q*, *K*, and *V* as:

(6) $$Q=W_{q}Y+bias_{q},\;$$

(7) $$K=W_{k}X+bias_{k},\;$$

(8) $$V=W_{v}X+bias_{v},\;$$

where $bias_{q},\;bias_{k}$ and $bias_{v}$ are the learnable biases. After generating the matrixes *Q*, *K*, and *V*, we computed the attention function and obtained the aligned feature $Z\in {\mathbb{R}}^{N\times d_{t}}$, as illustrated in Figure 4.

### 3.5. Feature Concatenation & Prediction

As shown in the rightmost box of Figure 4, we concatenated the aligned and point cloud features and sent them to the fully connected layer to obtain the final classification decision. Finally, the classification decision was normalized by the softmax function.

## 4. Results

In this section, we demonstrate the effectiveness of the proposed MTGEA components with the training and test sets of the DGUHA dataset. We performed all experiments on a machine with an Intel Xeon-Gold 6226 CPU, 192GB RAM (Intel Corporation, Santa Clara, CA, USA), and RTX 2080 Ti (Gigabyte, New Taipei City, Taipei) graphic card. We report the accuracy and weighted F1 score value as the evaluation metrics. The weighted F1 score is one of the metrics that take imbalanced data into account. Originally, the F1 score was calculated as follows:

(9) $$F_{1}\;score=\frac{2\cdot Recall\cdot Precision}{Recall+Precision},\;$$

where Recall is True Positive/True Positive + False Negative, and Precision is True Positive/True Positive + False Positive. We considered the weighted F1 score so that the ratio of the classes was balanced. (Approximately, running: 0.1432, jumping: 0.1419, sitting down and standing up: 0.1419, both upper limb extension: 0.1432, falling forward: 0.1432, right limb extension: 0.1432, and left limb extension: 0.1432.)

Three MTGEA models were trained using the three augmented types of data. We trained each model with a batch size of 13 for 300 epochs and used stochastic gradient descent with a learning rate of 0.01. Then, we froze the weights of the Kinect stream to verify the possibility of human activity recognition using radar data only. Therefore, only the test dataset of the point cloud was fed into the network during the test process, and the results are shown in Table 2.

**Figure 5:**
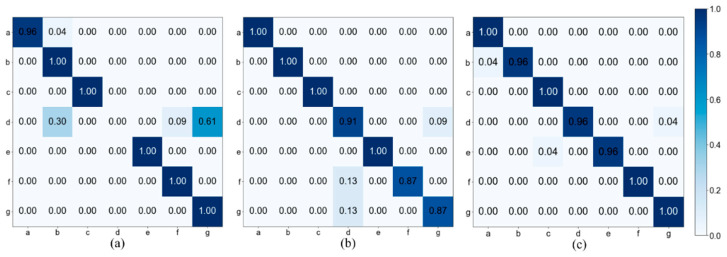
Confusion matrixes of three MTGEA models with different augmented data. (**a**) MTGEA (ZP + Skeleton), (**b**) MTGEA (GN + Skeleton), and (**c**) MTGEA (AHC + Skeleton).

Among the three augmented point cloud datasets, the MTGEA model that used the ZP augmentation strategy for sparse point clouds performed poorly in terms of prediction since the missing points were replaced by zeros only. However, the other models using multiple different augmentation strategies achieved higher accuracies of around 90%. In our evaluation, the best-performing MTGEA model, which was the one that used the AHC augmentation strategy, achieved a test accuracy of 98.14% and a weighted F1 score of 98.14%. This was 13.05% higher than the accuracy of the MTGEA model that used the ZP augmentation strategy and 3.11% higher than that using the GN augmentation strategy. This result indicates that the AHC algorithm can augment sparse point clouds more effectively. The confusion matrixes for the visualization of classification performance for our DGUHA dataset are illustrated in Figure 5, and the a–g labels denote the seven types of activity shown in Figure 2. According to the confusion matrix in Figure 5c, the MTGEA model that used the AHC augmentation strategy classified (a) running, (c) sitting down and standing up, (f) right limb extension, and (g) left limb extension 100% correctly. However, a few activities were confused with other activities; these were (b) jumping, (d) both upper limb extension, and (e) falling forward. However, these activities still achieved a high accuracy of over 95%.

According to the confusion matrix in Figure 5b, the MTGEA model that used the GN augmentation strategy achieved an accuracy under 95% for three out of seven activities. The three activities, (d) both upper limb extension, (f) right limb extension, and (g) left limb extension, are somewhat similar, as the arms or arms and legs moved away from the body and then moved back toward the body.

The MTGEA model that used the ZP augmentation strategy achieved 0% accuracy for (d) both upper limb extension activity, as this activity was somewhat confused with (b) jumping, (f) right limb extension, and (g) left limb extension, as shown in Figure 5a.

From these observations, we found that simple movements in which the body remains still and only the arms or legs move are generally harder to recognize than complex movements requiring the whole body, such as moving from left to right or running. Finally, the MTGEA model that used the AHC augmentation strategy achieved 95% accuracy for all activities, indicating the robustness of the model for simple activities that do not have complex movements distinct from other activities.

In addition, ablation studies were performed to demonstrate the necessity of the multimodal framework and attention mechanism in the proposed model.

## 5. Ablation Studies

### 5.1. Ablation Study for the Multimodal Framework

Ablation experiments were performed to justify the multimodal design of the proposed model. Single-modal models were created using a one-stream ST-GCN, and the ST-GCN architecture was the same as that of the MTGEA. The accuracy and weighted F1 score of the single-modal models are shown in Table 3. Compared to the multimodal models with the same augmented data, the single-modal models generally showed lower performance.

In the case of point clouds, the single-modal model used augmented point clouds with ZP and achieved 81.99% accuracy and a weighted F1 score of 81.51%. This was 3.1% lower in accuracy than the MTGEA model that used ZP. Notably, however, the single-modal model achieved a 2.16% higher weighted F1 score, as it classified (d) both upper limb extension activities 57% correctly. However, it classified the remaining activities incorrectly more often than the MTGEA model.

The second single-modal model that used augmented point clouds with GN achieved 92.55% accuracy and a weighted F1 score of 92.45%. These were 2.48% and 2.68% lower, respectively, than those of the MTGEA model that used the GN. The third single modal model used augmented point clouds with AHC and achieved 93.79% accuracy and a weighted F1 score of 93.80%, and both values were over 4% lower than those of the MTGEA.

The single-modal model that used skeleton data showed the best performance in this ablation experiment. It achieved an accuracy of 97.52% and a weighted F1 score of 97.51%, which were only 0.62% and 0.63% lower, respectively, than those of the MTGEA model that used the AHC augmentation strategy. These results seem to imply that since two useful datasets could be exploited by a multimodal framework, the multimodal models’ performance was generally better than that of the single-modal models’.

### 5.2. Ablation Study for the Attention Mechanism

**Figure 6:**
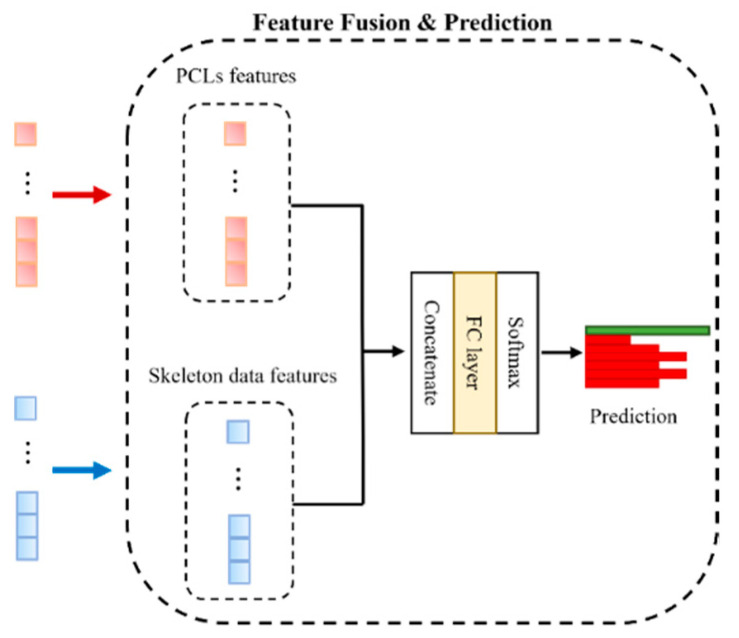
Multimodal feature fusion by concatenation. After features were extracted by three ST-GCN layers, the point cloud and skeleton data features were concatenated and fed into the fully connected layer. Then, a softmax classifier made a prediction.

Ablation experiments without an attention mechanism were conducted. Many feature fusion strategies have been studied to combine features effectively, and concatenation is one of the most popular methods. In this experiment, we concatenated two feature representations extracted by the ST-GCN before sending them to the fully connected layer instead of the attention mechanism, as illustrated in Figure 6. Then, we fed them to a softmax classifier to form a prediction.

Table 4 describes the results, which reveal the necessity of an attention mechanism. The best-performing MTGEA model achieved 98.14% accuracy, whereas the MTGEA model without attention that used the same multimodal two-stream framework achieved a lower accuracy of 96.27%. The weighted F1 score was also 1.9% lower than the MTGEA model with attention.

In the case of the MTGEA model without attention that used the GN augmentation strategy, it had a 0.62% lower accuracy and a 0.73% lower weighted F1 score than the original MTGEA model with the same augmentation strategy. Similarly, the MTGEA model without attention that used the ZP augmentation strategy had a 1.24% lower accuracy and a 1.58% lower weighted F1 score than the original MTGEA model that used the ZP augmentation strategy.

One notable point is that the MTGEA model without an attention mechanism generally had higher score values than the single-modal models, except for one weighted F1 score, while displaying lower score values than the MTGEA model with an attention mechanism. This means that utilizing accurate skeletal features from the Kinect sensor was critical. Additionally, comparisons between models with the same multimodal two-stream framework but with and without an attention mechanism indicated the necessity of an attention mechanism.

## 6. Conclusions

**Figure 7:**
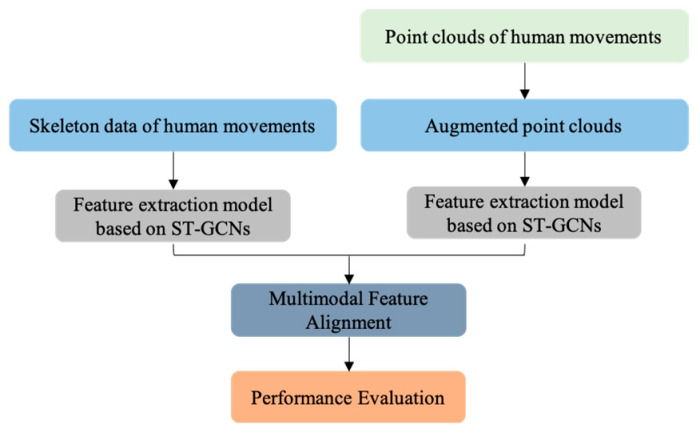
Proposed model diagram.

This paper presented a radar-based human activity recognition system called MTGEA that does not cause an invasion of privacy or require strict lighting environments. The proposed MTGEA model can classify human activities in a 3D space. To improve the accuracy of human activity recognition using sparse point clouds only, MTGEA uses a multimodal two-stream framework with the help of accurate skeletal features obtained from Kinect models. We used an attention mechanism for efficient multimodal data alignment. Moreover, we provided a newly produced dataset, called the DGUHA, that contains human skeleton data from a Kinect V4 sensor and 3D coordinates from a mmWave radar sensor. MTGEA was evaluated extensively using the DGUHA dataset. The results obtained after training the MTGEA model show that the proposed MTGEA model successfully recognizes human activities using sparse point clouds alone. Training/test datasets, including the raw dataset of DGUHA, are provided on our GitHub page. An ablation study on the multimodal two-stream framework was conducted, and it showed that two-stream framework structures were better than single-modal framework structures for human activity recognition. A similar conclusion was drawn from the second ablation study. This is because even when comparing the results with the MTGEA model that did not consist of an attention mechanism, it showed better performance than the single-modal framework structure. The second ablation study shows the effectiveness of an attention mechanism, an alignment method we used to leverage accurate skeletal features. For the same augmented point clouds, the MTGEA model without an attention mechanism had lower score values than that with an attention mechanism. In this experiment, we chose concatenation as a feature fusion strategy. Our experimental evaluations show the efficiency and necessity of each component of our MTGEA model. The MTGEA uses a multimodal two-stream framework to address the sparse point clouds and an attention mechanism to consider efficient alignment for two multimodal datasets. The entire workflow diagram is shown in Figure 7. Although the model needs some improvement for distinguishing simple activities that do not have complex movements, it can be one of the first steps toward creating a smart home care system.

## Figures and Tables

**Table 1:** Descriptive statistics for data samples.

Activity	Mean	Median	Mode	Min	Max
Running	12.79	13.0	13	3	24
Jumping	9.65	10.0	10	3	3
Sitting down and standing up	5.86	6.0	5	2	13
Both upper limb extension	6.18	5.0	3	2	17
Falling forward	3.78	4.0	3	2	8
Right limb extension	6.11	6.0	5	2	13
Left limb extension	5.22	5.0	5	2	13

**Table 2:** Test Accuracy on the DGUHA dataset.

Model	Accuracy (%)	Weighted F_1_ Score (%)
MTGEA (ZP + Skeleton)	85.09	79.35
MTGEA (GN + Skeleton)	95.03	95.13
MTGEA (AHC + Skeleton)	98.14	98.14

**Table 3:** Performance comparison of single-modal models on the DGUHA dataset.

Model	Accuracy (%)	Weighted F_1_ Score (%)
Augmented point clouds using ZP	81.99	81.51
Augmented point clouds using GN	92.55	92.45
Augmented point clouds using AHC	93.79	93.80
Skeleton data	97.52	97.51

**Table 4:** Performance comparison of fusion models on the DGUHA dataset.

Model	Accuracy (%)	Weighted F_1_ Score (%)
MTGEA (ZP + Skeleton) without attention	83.85	77.77
MTGEA (GN + Skeleton) without attention	94.41	94.40
MTGEA (AHC + Skeleton) without attention	96.27	96.24

## Data Availability

Publicly available datasets were used in this study. The datasets can be found here: (1) MMActivity (https://github.com/nesl/RadHAR accessed on 1 March 2023) and (2) ours: https://github.com/AIC-DGU/MTGEA (accessed on 1 March 2023).

## References

[1] Vaiyapuri T., Lydia E.L., Sikkandar M.Y., Diaz V.G., Pustokhina I.V., Pustokhin D.A. (2021). Internet of Things and Deep Learning Enabled Elderly Fall Detection Model for Smart Homecare. IEEE Access.

[2] Ma W., Chen J., Du Q., Jia W. PointDrop: Improving object detection from sparse point clouds via adversarial data augmentation. Proceedings of the 2020 25th International Conference on Pattern Recognition (ICPR).

[3] Xu S., Zhou X., Ye W., Ye Q. (2022). Classification of 3D Point Clouds by a New Augmentation Convolutional Neural Network. IEEE Geosci. Remote Sens. Lett..

[4] Kim K., Kim C., Jang C., Sunwoo M., Jo K. (2021). Deep learning-based dynamic object classification using LiDAR point cloud augmented by layer-based accumulation for intelligent vehicles. Expert Syst. Appl..

[5] Kulawiak M. (2022). A Cost-Effective Method for Reconstructing City-Building 3D Models from Sparse Lidar Point Clouds. Remote Sens..

[6] Singh A.D., Sandha S.S., Garcia L., Srivastava M. Radhar: Human activity recognition from point clouds generated through a millimeter-wave radar. Proceedings of the 3rd ACM Workshop on Millimeter-Wave Networks and Sensing Systems.

[7] Palipana S., Salami D., Leiva L.A., Sigg S. (2021). Pantomime: Mid-air gesture recognition with sparse millimeter-wave radar point clouds. Proc. ACM Interact. Mob. Wearable Ubiquitous Technol..

[8] Vonstad E.K., Su X., Vereijken B., Bach K., Nilsen J.H. (2020). Comparison of a deep learning−based pose estimation system to marker−based and kinect systems in exergaming for balance training. Sensors.

[9] Radu I., Tu E., Schneider B. (2020). Relationships between body postures and collaborative learning states in an Augmented Reality Study. International Conference on Artificial Intelligence in Education, Ifrane, Morocco, 6–10 July 2020.

[10] Shahroudy A., Liu J., Ng T.-T., Wang G. NTU RGB+D: A Large Scale Dataset for 3D Human Activity Analysis. Proceedings of the IEEE Conference on Computer Vision and Pattern Recognition 2016, Las Vegas Valley.

[11] Liu J., Shahroudy A., Perez M., Wang G., Duan L.Y., Kot A.C. (2019). NTU RGB+D 120: A large-scale benchmark for 3D human activity understanding. IEEE Trans. Pattern Anal. Mach. Intell..

[12] Haocong R., Shihao X., Xiping H., Jun C., Bin H. (2021). Augmented skeleton based contrastive action learning with momentum LSTM for unsupervised action recognition. Inf. Sci..

[13] Ryselis K., Blažauskas T., Damaševičius R., Maskeliūnas R. (2022). Computer-aided depth video stream masking framework for human body segmentation in depth sensor images. Sensors.

[14] Wozniak M., Wieczorek M., Silka J., Polap D. (2021). Body pose prediction based on motion sensor data and recurrent neural network. IEEE Trans. Ind. Inform..

[15] Weiyao X., Muqing W., Min Z., Ting X. (2021). Fusion of skeleton and RGB features for RGB-D human action recognition. IEEE Sens. J..

[16] Zheng C., Feng J., Fu Z., Cai Y., Li Q., Wang T. Multimodal relation extraction with efficient graph alignment. Proceedings of the MM ’21: ACM Multimedia Conference.

[17] Yang W., Zhang J., Cai J., Xu Z. (2021). Shallow graph convolutional network for skeleton-based action recognition. Sensors.

[18] Ogundokun R.O., Maskeliūnas R., Misra S., Damasevicius R. (2022). Hybrid inceptionv3-svm-based approach for human posture detection in health monitoring systems. Algorithms.

[19] Sengupta A., Cao S. (2021). mmPose-NLP: A natural language processing approach to precise skeletal pose estimation using mmwave radars. arXiv.

[20] Lee G., Kim J. (2022). Improving human activity recognition for sparse radar point clouds: A graph neural network model with pre-trained 3D human-joint coordinates. Appl. Sci..

[21] Pan L., Chen X., Cai Z., Zhang J., Liu Z. Variational Relational Point Completion Network. Proceedings of the 2021 IEEE/CVF Conference on Computer Vision and Pattern Recognition (CVPR).

[22] Zhang R., Cao S. (2019). Real-time human motion behavior detection via CNN using mmWave radar. IEEE Sens. Lett..

[23] Yan S., Xiong Y., Lin D. (2018). Spatial temporal graph convolutional networks for skeleton-based action recognition. arXiv.

[24] Kipf T.N., Welling M. Semi-supervised classification with graph convolutional networks. Proceedings of the 5th International Conference on Learning Representations.

[25] Bahdanau D., Cho K.H., Bengio Y. Neural machine translation by jointly learning to align and translate. Proceedings of the 3rd International Conference on Learning Representations.

[26] Rashid M., Khan M.A., Alhaisoni M., Wang S.-H., Naqvi S.R., Rehman A., Saba T. (2020). A Sustainable Deep Learning Framework for Object Recognition Using Multi-Layers Deep Features Fusion and Selection. Sustainability.

[27] Yen C.-T., Liao J.-X., Huang Y.-K. (2021). Feature Fusion of a Deep-Learning Algorithm into Wearable Sensor Devices for Human Activity Recognition. Sensors.

[28] Wu P., Cui Z., Gan Z., Liu F. (2020). Three-Dimensional ResNeXt Network Using Feature Fusion and Label Smoothing for Hyperspectral Image Classification. Sensors.

[29] Petrovska B., Zdravevski E., Lameski P., Corizzo R., Štajduhar I., Lerga J. (2020). Deep learning for feature extraction in remote sensing: A case-study of aerial scene classification. Sensors.

[30] Vaswani A., Shazeer N., Parmar N., Uszkoreit J., Jones L., Gomez A.N., Kaiser L., Polosukhin I. (2017). Attention is all you need. arXiv.

